# Perioperative prothrombin time activity trajectories and subsequent mortality after surgical repair of acute type A aortic dissection

**DOI:** 10.3389/fmed.2026.1885240

**Published:** 2026-10-19

**Authors:** Hongle Yan, Xuejun Shen, Huozhen Weng, Weiping Li, Shengli Yang

**Affiliations:** 1 Clinical Research Center, First Affiliated Hospital of Shantou University Medical College, Shantou, Guangdong, China; 2 Department of Clinical Medicine, Shantou University Medical College, Shantou, Guangdong, China

**Keywords:** acute type A aortic dissection, group-based trajectory modeling, landmark analysis, longitudinal trajectory, mortality, prothrombin time activity

## Abstract

**Background:**

Perioperative prothrombin time activity (PTA) changes rapidly during surgical repair of acute type A aortic dissection (ATAAD), but single measurements do not capture heterogeneous longitudinal patterns. We sought to identify recurring perioperative PTA trajectories, characterize their clinical context, and examine their association with subsequent all-cause mortality.

**Methods:**

This single-center retrospective cohort included 284 adults who underwent ATAAD repair from 2021 through 2023. Group-based trajectory modeling used the last preoperative and observed postoperative day (POD) 1–7 PTA measurements. Patients required at least three observed PTA measurements for trajectory modeling. Mortality analyses began immediately after POD7. The primary Cox model adjusted for age and sex; Firth regression addressed sparse events. Incremental information beyond last preoperative PTA was evaluated by a prespecified likelihood-ratio comparison.

**Results:**

The trajectory cohort comprised 268 patients with 1,895 observations and 27 deaths after POD7. Four perioperative PTA trajectory groups were identified: moderate-stable [87 (32.5%)], low-declining [50 (18.7%)], preserved-rapidly-declining [61 (22.8%)], and high-preserved [70 (26.1%)]. Cumulative mortality differed globally (log-rank *P* = 0.00210), and trajectory group remained associated with subsequent mortality in the age- and sex-adjusted Cox model (global *P* = 0.0322) and Firth sensitivity analysis (global *P* = 0.0481). The prespecified direct low-declining-versus-preserved-rapidly-declining comparison did not demonstrate a clear difference. Adding trajectory group to age, sex, and last preoperative PTA did not show clear incremental information (likelihood-ratio *P* = 0.304; Firth *P* = 0.380). After exclusion of mechanical-valve recipients, the principal trajectory structure was largely preserved, although this sensitivity analysis did not exclude treatment-related effects on PTA.

**Conclusion:**

Four reproducible perioperative PTA trajectories characterized distinct longitudinal patterns and were associated with mortality after the POD7 landmark. These trajectories should be interpreted as composite perioperative patterns reflecting both disease and treatment context rather than as intrinsic coagulation states. They were not ordered mortality-risk tiers and did not show clear incremental prognostic information beyond last preoperative PTA. External validation is required.

## 1 Introduction

Acute type A aortic dissection (ATAAD) is a cardiovascular emergency that requires prompt surgical repair, yet early mortality remains substantial even in contemporary practice (1–3). Hemostatic disturbance is a prominent component of this clinical course. The dissection process can expose tissue factor and activate coagulation and fibrinolysis before surgery, while cardiopulmonary bypass, hypothermia, circulatory arrest, operative trauma, hemorrhage, and transfusion can further perturb perioperative hemostasis (4–7). These overlapping disease- and treatment-related influences make coagulation status both clinically relevant and difficult to summarize with a single measurement. They also complicate interpretation: an abnormal result may reflect the presenting dissection, physiological stress, operative exposure, administered treatment, or a combination of these factors. A longitudinal description may therefore be more informative about perioperative heterogeneity than an isolated value, even when it cannot identify the underlying mechanism.

Prothrombin time activity (PTA) expresses a prothrombin-time result as a PT-derived percentage activity and therefore reflects the extrinsic/common-pathway measurement framework rather than a direct assay of any single coagulation factor (8). Its interpretation depends on the analytical system, including thromboplastin reagent and calibration, and values should not be assumed to be fully interchangeable among laboratories. In patients with ATAAD, preoperative coagulation abnormalities have been associated with disease severity and adverse outcomes, supporting careful study of routine coagulation markers while avoiding mechanistic claims that such assays cannot establish (4, 9, 10). PTA is repeatedly measured during perioperative care at many centers, creating an opportunity to examine how PTA changes over the perioperative course rather than relying only on an admission or preoperative value. Because PTA is a routine laboratory measure rather than a complete assessment of hemostasis, its clinical meaning should remain anchored to the broader coagulation, surgical, and treatment context.

Serial PTA measurements may follow different longitudinal morphologies. For example, patients who begin with similar postoperative values may have arrived there from markedly different preoperative levels, and patients with comparable preoperative values may diverge after surgery. A single value cannot represent these paths. Group-based trajectory modeling provides a descriptive framework for identifying latent groups with similar within-person patterns, provided that the number and shape of groups are selected with attention to fit, classification, stability, parsimony, and clinical interpretability (11–13). Existing ATAAD literature has characterized perioperative coagulopathy and individual biomarkers, but heterogeneous PTA evolution across the preoperative-to-postoperative window remains incompletely described. Describing such heterogeneity is a different objective from building a prognostic model: it asks whether recurring shapes can be identified and whether those shapes retain their structure under relevant sensitivity analyses.

We therefore aimed to identify reproducible perioperative PTA trajectories using the last preoperative and observed POD1–7 measurements, characterize the clinical and treatment context of the resulting trajectory groups, and examine their association with subsequent all-cause mortality using a POD7 landmark. Secondary objectives were to evaluate whether trajectory-group membership provided incremental prognostic information beyond the last preoperative PTA and to examine the structural stability of the trajectories after exclusion of mechanical-valve recipients. The study was designed to characterize longitudinal heterogeneity; it did not evaluate real-time classification, a PTA-guided intervention, or causal mechanisms.

## 2 Methods

### 2.1 Study design, setting, and participants

This single-center retrospective cohort study included consecutive adults with computed tomography angiography-confirmed acute type A aortic dissection who underwent surgical repair at the First Affiliated Hospital of Shantou University Medical College between January 2021 and December 2023. Hospital records identified 1,002 patients with aortic dissection; 522 had Stanford type A dissection, 444 met the acute definition, and 285 surgically treated ATAAD patients met the clinical eligibility criteria. One clinically eligible patient was excluded because essential perioperative laboratory/PTA source data were unavailable for reliable exposure ascertainment, leaving 284 patients in the final study cohort (Figure 1).

**Figure 1:**
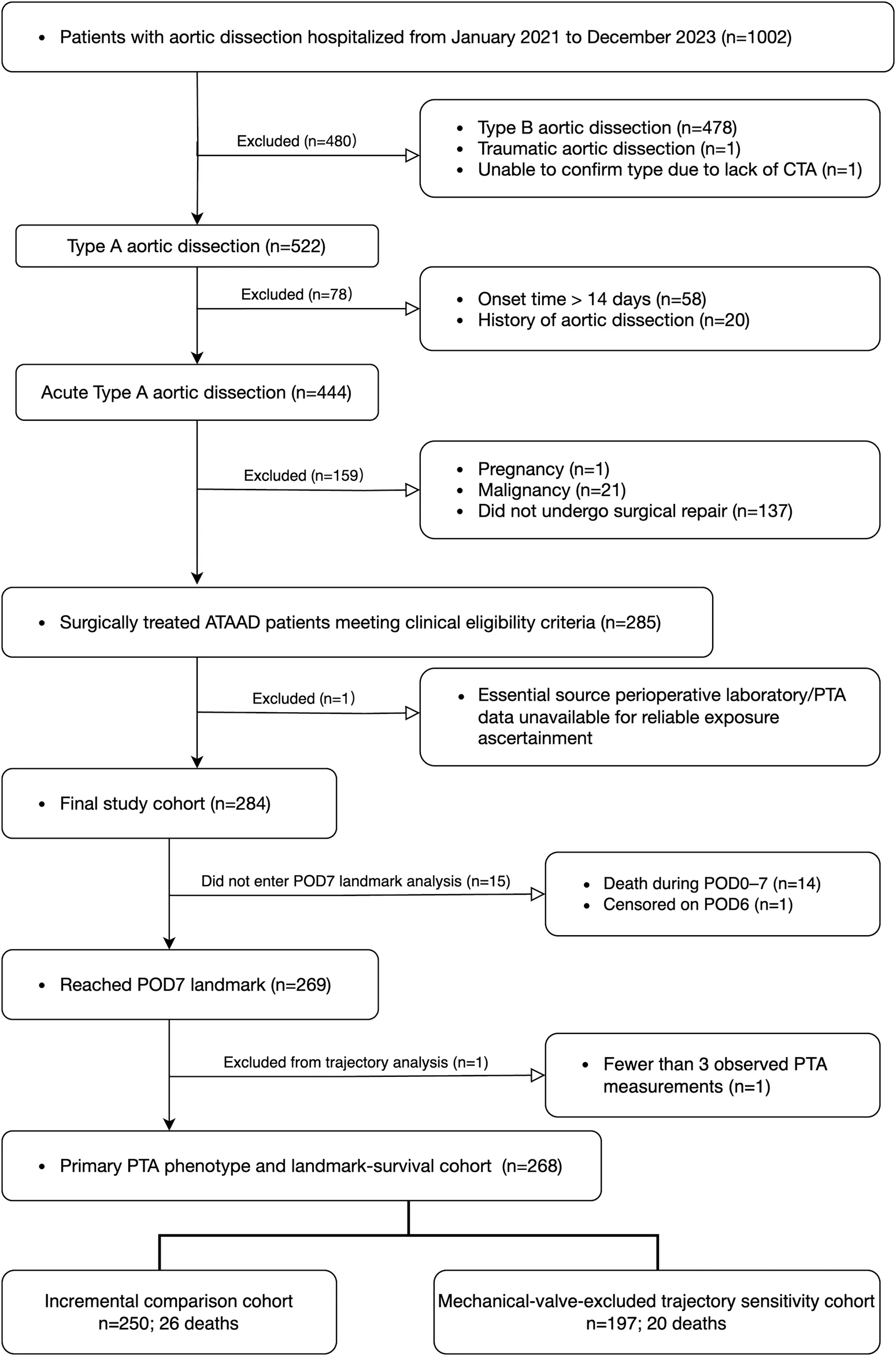
Study flow and analysis populations. Among 1,002 patients with aortic dissection hospitalized from January 2021 to December 2023, 480 were excluded (type B aortic dissection, *n* = 478; traumatic aortic dissection, *n* = 1; type not confirmable because CTA was unavailable, *n* = 1), leaving 522 with Stanford type A dissection. Seventy-eight were excluded (onset >14 days, *n* = 58; history of aortic dissection, *n* = 20), leaving 444 with acute type A dissection. A further 159 were excluded (pregnancy, *n* = 1; malignancy, *n* = 21; did not undergo surgical repair, *n* = 137), leaving 285 surgically treated ATAAD patients who met the clinical eligibility criteria. One was excluded because essential perioperative laboratory/PTA source data were unavailable for reliable exposure ascertainment, leaving 284 in the final study cohort. Fourteen patients died on POD0–7 and one was censored on POD6; 269 reached the POD7 landmark. One had fewer than three observed PTA measurements, leaving 268 in the primary trajectory-analysis and landmark-survival cohort. The incremental comparison included 250 patients with 26 deaths, and the mechanical-valve-excluded trajectory sensitivity cohort included 197 patients with 20 deaths. ATAAD, acute type A aortic dissection; CTA, computed tomography angiography; POD, postoperative day; PTA, prothrombin time activity.

The study was approved by the Ethics Committee of the First Affiliated Hospital of Shantou University Medical College (No. B-2022-229). The requirement for informed consent was waived owing to the retrospective nature of the study, and data were anonymized before analysis.

### 2.2 Data sources and temporal framework

Clinical, anatomical, operative, laboratory, treatment, and outcome data were obtained from the electronic medical records and study data set. Variables measured before surgery were treated as baseline prognostic descriptors. Intraoperative variables were treated as exposures that could influence postoperative PTA while also reflecting procedural complexity. Delayed sternal closure and other postoperative variables were not treated as conventional baseline confounders. Postoperative treatments and clinical consequences could occur in response to evolving illness and could also lie on pathways linking perioperative status to later outcome. Accordingly, comparisons across trajectory groups were descriptive, and these variable sets were not interpreted as determinants of trajectory membership or used to support causal claims.

Among the 268 patients assigned to a PTA trajectory group, operative durations, arch and proximal procedures, valve type, aortic regurgitation grade, primary-entry-tear location, and available preoperative CTA findings were compared using the observed denominator for each variable. Arch-repair extent was individualized according to dissection anatomy, malperfusion, overall clinical condition, and surgeon judgment. Preoperative shock, tamponade, and organ-specific malperfusion were abstracted from available clinical records and CTA reports and categorized as documented present, documented absent, ambiguous, or not documented. Clinical malperfusion syndromes, radiographic perfusion abnormalities, and anatomical branch involvement were considered separately.

### 2.3 PTA measurement, timing, and availability

Prothrombin time activity (PTA) was measured during routine clinical care in the institutional central laboratory and reported as a PT-derived percentage activity. The institutional lower reference limit was 70%. Detailed historical information on the analyzer, thromboplastin reagent, and calibration procedure could not be reliably recovered (8). The admission PTA was the first PTA recorded at admission. The last preoperative PTA was the last available measurement before surgery and defined trajectory time 0; it was not a postoperative-day-0 measurement.

Trajectory modeling used the last preoperative value and observed PTA values from postoperative days (PODs) 1–7. Sampling followed clinical care and was therefore irregular. When more than one measurement was available within a postoperative day, the documented day-level value in the analysis data set was used. Values recorded after discharge were retained when they fell within POD1–7; days without a recorded value remained missing. Unobserved day-level values were not interpolated, carried forward, or otherwise imputed. The availability profile is reported in Supplementary Table S1.

### 2.4 Outcome and follow-up

The outcome was all-cause mortality through POD365. Follow-up time was right-censored at the last date on which vital status was known and administratively capped at POD365. Causes of death were unavailable. Because some non-decedents had shorter observation, analyses did not assume complete 1-year follow-up.

### 2.5 Group-based trajectory modeling

The trajectory cohort was defined after the POD7 observation window. Fourteen patients who died on POD0–7 and one patient censored on POD6 could not enter a post-POD7 landmark analysis, leaving 269 patients observable at the landmark. The final eligibility rule required at least three observed PTA measurements. One patient did not meet this criterion; 268 patients therefore contributed to group-based trajectory modeling (GBTM). Because trajectory-group assignment used measurements through POD7, it was available only after completion of the POD7 observation window and not at surgery. The censored-normal model implemented in the trajeR package used the last preoperative value at time 0 and available POD1–7 PTA values (11–13).

Models with two to five groups were fitted with multiple starting values. The primary optimized four-group model used polynomial orders 2, 3, 3, and 2 and group-specific residual standard deviations (ssigma = FALSE). Selection considered Akaike and Bayesian information criteria, average posterior probabilities (AvePP), odds of correct classification (OCC), entropy, group proportions, individual posterior probabilities, bootstrap stability, and agreement in a high-completeness subgroup. The optimized three- and five-group models were retained as structural sensitivity analyses. The five-group model improved information criteria but added a level split and showed poorer bootstrap stability. The four-group solution was selected before opening survival outcomes because it balanced fit, classification, distinct longitudinal morphology, stability, and parsimony. Patients were assigned to the group with the largest posterior probability.

### 2.6 Post-POD7 landmark survival analysis

Survival analyses began immediately after POD7 to preserve temporal ordering between the exposure window and subsequent outcome (14, 15). Analysis time was min(observed survival time, 365)−7, with a maximum of 358 days, and events were deaths after POD7 through POD365. Cumulative mortality was estimated by the Kaplan–Meier method, and trajectories were compared with a global log-rank test. The primary Cox model included trajectory group, age, and sex. This parsimonious adjustment was prespecified in view of 27 post-landmark deaths and was not intended to estimate causal effects. Proportional-hazards assumptions were assessed using Schoenfeld residuals.

Because the high-preserved reference group had one death, the primary inferential target was the global association between trajectory group and subsequent mortality. The final Cox model contained five regression parameters (three trajectory indicators, age, and sex); no data-driven candidate-variable screening was used. Class-specific hazard ratios were treated as secondary and imprecise. A direct G2-versus-G3 contrast was specified before the survival outcomes were examined because the low-declining and preserved-rapidly-declining groups represented distinct longitudinal morphologies. Firth-penalized Cox regression was used as a sparse-data sensitivity analysis (16).

### 2.7 Incremental information beyond the last preoperative PTA

The incremental analysis included 250 landmark participants with a recorded last preoperative PTA and 26 deaths. Four nested models were compared: M0, age and sex; M1, M0 plus last preoperative PTA; M2, M0 plus the four-group trajectory variable; and M3, M0 plus both last preoperative PTA and trajectory group. The prespecified primary comparison was M1 versus M3 using a three-degree-of-freedom likelihood-ratio test; the corresponding Firth penalized-likelihood comparison was a sensitivity analysis. Harrell C, Uno C, the time-dependent area under the curve at day 358 after the landmark, Brier score, and calibration slope were estimated with 500 bootstrap repetitions and reported after optimism correction. Decision-curve analysis was not performed because no individual-level prediction model or clinical decision threshold was specified. The study did not evaluate a PTA-guided intervention.

### 2.8 Sensitivity and complementary analyses

The functional form of last preoperative PTA was evaluated before retaining a linear term. Simple PTA analyses used a common complete-case cohort of 249 patients with both last preoperative and POD1 PTA available and 26 post-landmark deaths. Model S0 included age, sex, and last preoperative PTA; S1 included age, sex, and POD1 PTA; and S2 included age, sex, last preoperative PTA, and the change from last preoperative PTA to POD1 (POD1 PTA minus last preoperative PTA). The primary change comparison was S0 versus S2. No *post hoc* POD3 or POD7 summary analysis was undertaken.

Available treatment-related data included proximal aortic procedure, prosthetic-valve status, and intraoperative red blood cell, plasma, platelet, and cryoprecipitate quantities. To examine whether the trajectory structure was sensitive to mechanical-valve receipt, trajectories were refitted without outcome information after excluding mechanical-valve recipients. Exclusion of all prosthetic-valve recipients was retained as a secondary structural analysis without survival-outcome assessment.

A complementary flexible joint longitudinal-survival model was reconstructed to examine continuously updated PTA, with prespecified convergence criteria. Because the primary model and prespecified simplified baseline-hazard rescue specifications failed to meet MCMC convergence criteria, no joint-model association estimate was retained for inference (17).

### 2.9 Statistical reporting

Continuous variables are reported as mean (standard deviation) or median [25th, 75th percentiles], and categorical variables as number (percentage), using observed denominators. Global descriptive comparisons used variable-specific one-way analysis of variance or Kruskal–Wallis tests for continuous variables and Pearson chi-square or Fisher exact Monte Carlo tests for categorical variables, as specified in the table footnotes. Perioperative continuous variables were compared using Kruskal–Wallis tests; categorical variables used Pearson chi-square tests when all expected cell counts were at least five and otherwise Fisher–Freeman–Halton Monte Carlo tests. These comparisons were two-sided, descriptive, and unadjusted for multiplicity; no pairwise comparisons were performed. No missing data were imputed. Analyses were performed in R version 4.4.0.

## 3 Results

### 3.1 Study population and PTA availability

Among 1,002 patients with aortic dissection hospitalized from January 2021 to December 2023, 522 had Stanford type A dissection, 444 met the acute definition, and 285 surgically treated ATAAD patients met the clinical eligibility criteria. One was excluded because essential perioperative laboratory/PTA source data were unavailable for reliable exposure ascertainment; the final study cohort therefore comprised 284 patients (Figure 1; Supplementary Table S9). The data set contained 4,037 PTA measurements, with a median of 12 [8–18] measurements per patient (range, 1–91). Across the cohort, observed PTA times ranged from 0 to 108 days relative to the last preoperative measurement.

Fourteen patients died during POD0–7 and one was censored on POD6, leaving 269 patients observable immediately after POD7. Of these, one had insufficient longitudinal information for trajectory assignment, yielding a trajectory cohort of 268 patients with 1,895 observed values across the last-preoperative/POD1–7 trajectory window. Among the 269 landmark-eligible patients, 136 (50.6%) had observations at all eight trajectory time points and 133 had at least one unobserved time point. All 268 trajectory participants had at least three observed PTA measurements and were eligible for trajectory modeling. Last preoperative PTA was available for 250 (92.9%); availability on POD1–7 was 268 (99.6%), 259 (96.3%), 255 (94.8%), 238 (88.5%), 223 (82.9%), 207 (77.0%), and 196 (72.9%), respectively (Supplementary Table S1). No unobserved day-level value was imputed.

Forty-two deaths occurred by POD365 in the final cohort: 14 on POD0–7, 20 on POD8–30, five on POD31–90, two on POD91–180, and one on POD181–365. Thirty-one deaths occurred in hospital and 11 after discharge. Among non-decedents, 113 were censored before POD365. In the landmark cohort, observed follow-up after POD7 ranged from 1 to 358 days. Cause-specific mortality could not be described because causes of death were unavailable. Follow-up, death-timing, and censoring details are reported in Supplementary Table S9.

### 3.2 Identification and internal validation of four PTA trajectory groups

The optimized four-group model identified moderate-stable (group 1; *n* = 87, 32.5%), low-declining (group 2; *n* = 50, 18.7%), preserved-rapidly-declining (group 3; *n* = 61, 22.8%), and high-preserved (group 4; *n* = 70, 26.1%) trajectory groups (Figure 2). Entropy was 0.830. Group-specific AvePP values were 0.897, 0.913, 0.898, and 0.922; corresponding OCC values were 18.33, 46.53, 28.92, and 33.89. The proportions with individual posterior probability ≥0.80 were 75.9%, 82.0%, 77.0%, and 85.7%.

**Figure 2:**
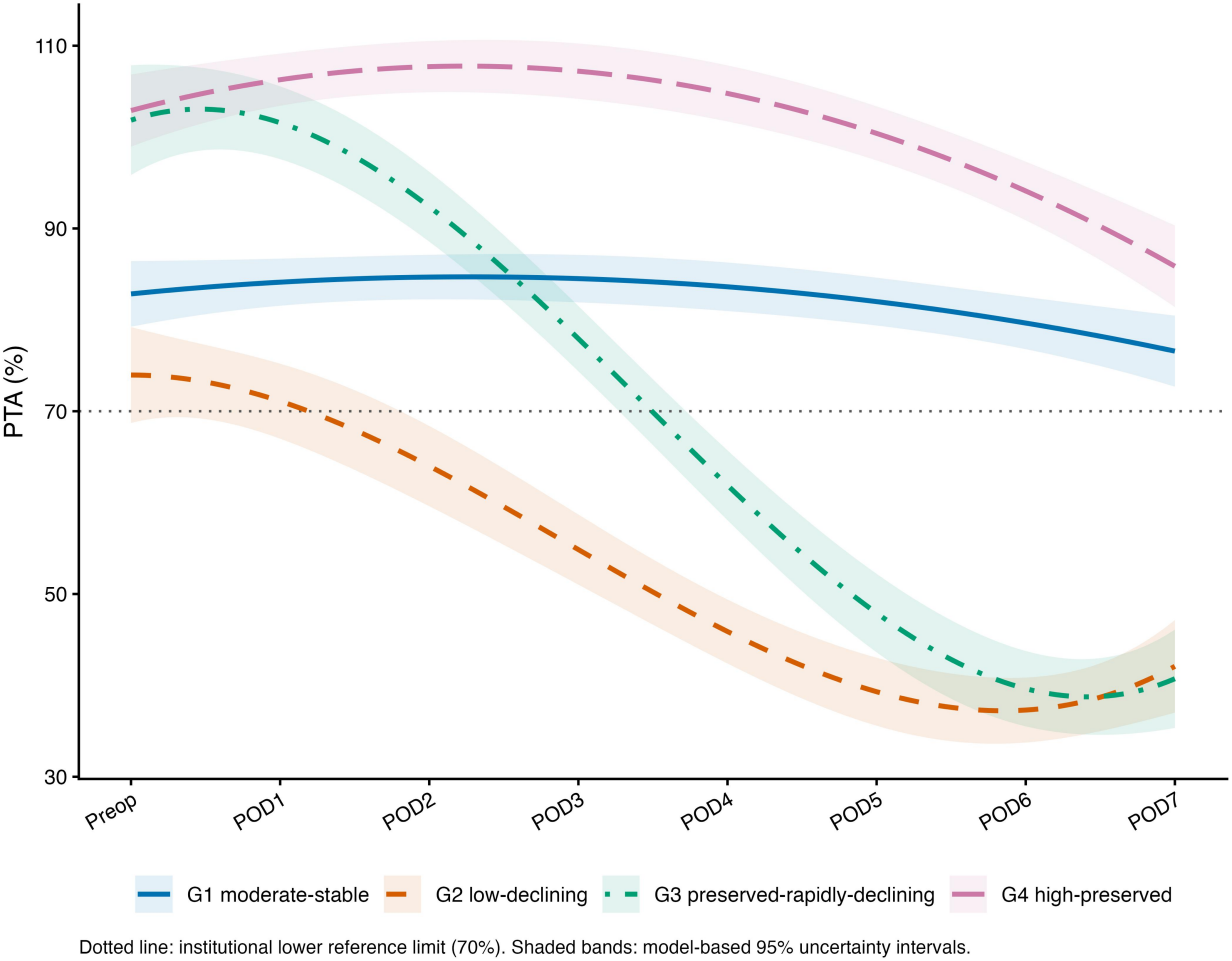
Four-group perioperative PTA trajectories. Model-based mean PTA trajectories are shown from the last preoperative measurement (Preop; time 0) through observed POD1–7 measurements for the moderate-stable, low-declining, preserved-rapidly-declining, and high-preserved trajectory groups. Shaded bands are validated model-based 95% uncertainty intervals. The horizontal dotted line at 70% marks the institutional lower reference limit. No interpolation, last-observation-carried-forward procedure, or imputation was used for unobserved day-level values. POD, postoperative day; PTA, prothrombin time activity.

The optimized K4 model had an AIC of 16,462.3 and BIC of 16,537.7, and all 30 multistart fits reached the same optimum. Although K5 had lower AIC and BIC (16,394.8 and 16,481.0), it introduced an additional level split and was less stable under bootstrap resampling: among 200 successful repetitions, K4 had five unstable and no collapsed solutions, whereas K5 had 23 unstable and three collapsed solutions. K3 had two unstable and no collapsed solutions. In the high-completeness subgroup (*n* = 235), agreement with primary K4 assignment was 98.7% [adjusted Rand index (ARI), 0.967; normalized mutual information, 0.953]. These outcome-blind diagnostics supported selection of K4 as the primary solution (Supplementary Tables S2–S4).

### 3.3 Clinical and perioperative characteristics

Clinical and perioperative characteristics of the 268 trajectory participants are summarized in Table 1, with further operative, anatomical, proximal-procedure, and plasma findings in Supplementary Table S10. Aortic cross-clamp duration differed across trajectory groups (global Kruskal–Wallis *P* = 0.004), whereas the corresponding *P* values were 0.205 for circulatory-arrest duration and 0.095 for total operation duration. Total arch replacement accounted for 243 of 267 classifiable arch procedures. Descriptively, Bentall procedures and mechanical valves were more frequent in the two declining trajectory groups than in the moderate-stable and high-preserved groups. Intraoperative plasma amount did not show a clear global difference across trajectory groups (*P* = 0.108). Primary-entry-tear location was available in 263 patients, including 115 with an ascending-aortic tear (Supplementary Table S10).

**Table 1:** Clinical and perioperative characteristics by perioperative PTA trajectory group.

Variable/level	Overall	Moderate-stable (*n* = 87)	Low-declining (*n* = 50)	Preserved-rapidly-declining (*n* = 61)	High-preserved (*n* = 70)	Global P	Available n	Missing n
Demographics								
Age, years	55.69 ± 11.37	58.32 ± 9.24	57.90 ± 13.85	53.66 ± 11.73	52.61 ± 10.59	0.003	268	0
Male sex: Yes	192 (71.6)	58 (66.7)	38 (76.0)	43 (70.5)	53 (75.7)	0.544	268	0
Body mass index, kg/m^2^	24.92 ± 3.43	24.82 ± 3.58	23.90 ± 2.91	24.51 ± 3.44	26.14 ± 3.28	0.002	268	0
Comorbidities								
Current/ever smoking: Yes	165 (61.6)	51 (58.6)	30 (60.0)	37 (60.7)	47 (67.1)	0.726	268	0
Hypertension: Yes	218 (81.3)	70 (80.5)	38 (76.0)	48 (78.7)	62 (88.6)	0.298	268	0
Diabetes mellitus: Yes	26 (9.7)	8 (9.2)	8 (16.0)	2 (3.3)	8 (11.4)	0.125	268	0
Chronic kidney disease: Yes	22 (8.2)	8 (9.2)	4 (8.0)	3 (4.9)	7 (10.0)	0.761	268	0
Coronary heart disease: Yes	17 (6.3)	2 (2.3)	3 (6.0)	6 (9.8)	6 (8.6)	0.189	268	0
Admission/preoperative								
Admission systolic blood pressure, mmHg	148.12 ± 33.49	152.61 ± 34.58	139.00 ± 28.15	147.16 ± 37.92	149.87 ± 30.78	0.138	268	0
Admission diastolic blood pressure, mmHg	82.41 ± 19.86	86.10 ± 19.67	76.42 ± 18.97	80.77 ± 21.84	83.51 ± 18.08	0.041	268	0
Admission PTA, %	93.14 ± 18.23	88.23 ± 14.61	80.35 ± 19.16	97.18 ± 15.85	104.85 ± 15.46	<0.001	268	0
Last preoperative PTA, %	92.21 ± 20.79	84.85 ± 14.16	74.37 ± 17.68	104.29 ± 18.69	104.30 ± 18.05	<0.001	250	18
Laboratory								
Platelet count, × 10^9^/L	169.00 [138.00, 204.00]	161.00 [137.50, 195.50]	153.00 [122.25, 202.75]	179.00 [151.00, 210.00]	178.50 [145.75, 204.00]	0.102	268	0
Fibrinogen, g/L	2.55 [1.97, 3.43]	2.54 [2.03, 3.24]	2.32 [1.85, 3.59]	2.59 [2.04, 3.48]	2.67 [1.97, 3.18]	0.888	268	0
Cholinesterase, kU/L	7.16 ± 1.80	7.10 ± 1.72	6.38 ± 1.77	7.28 ± 1.59	7.71 ± 1.91	<0.001	268	0
Creatinine, μmol/L	105.00 [80.36, 138.01]	105.00 [80.74, 133.50]	112.46 [79.11, 150.66]	112.00 [83.11, 142.00]	98.65 [79.33, 135.85]	0.588	268	0
NT-proBNP, pg/mL	443.65 [164.80, 944.33]	439.30 [181.00, 928.65]	649.40 [355.98, 1,981.93]	450.40 [154.80, 925.10]	275.50 [101.10, 719.02]	0.002	268	0
Aortic/anatomical								
Primary entry tear location: ascending aorta	115 (43.7)	27 (32.1)	33 (66.0)	35 (57.4)	20 (29.4)	<0.001	263	5
aortic arch/arch branches	70 (26.6)	30 (35.7)	9 (18.0)	13 (21.3)	18 (26.5)		263	5
descending aorta	36 (13.7)	13 (15.5)	4 (8.0)	3 (4.9)	16 (23.5)		263	5
no definite primary entry identified	42 (16.0)	14 (16.7)	4 (8.0)	10 (16.4)	14 (20.6)		263	5
Ascending aortic diameter, cm	4.50 [4.50, 5.00]	4.50 [4.50, 5.00]	5.00 [4.50, 5.50]	5.00 [4.50, 5.00]	4.50 [4.50, 5.00]	<0.001	245	23
Pericardial effusion: none/not documented	31 (11.6)	12 (13.8)	2 (4.0)	5 (8.2)	12 (17.1)	0.196	268	0
mild	127 (47.4)	38 (43.7)	20 (40.0)	35 (57.4)	34 (48.6)		268	0
moderate	72 (26.9)	24 (27.6)	17 (34.0)	14 (23.0)	17 (24.3)		268	0
large/severe	38 (14.2)	13 (14.9)	11 (22.0)	7 (11.5)	7 (10.0)		268	0
Aortic regurgitation: none/not documented	24 (9.0)	12 (13.8)	4 (8.0)	2 (3.3)	6 (8.6)	<0.001	268	0
mild	117 (43.7)	46 (52.9)	11 (22.0)	20 (32.8)	40 (57.1)		268	0
moderate	58 (21.6)	15 (17.2)	13 (26.0)	13 (21.3)	17 (24.3)		268	0
severe	69 (25.7)	14 (16.1)	22 (44.0)	26 (42.6)	7 (10.0)		268	0
Surgery								
Proximal aortic procedure: Bentall procedure	90 (33.6)	13 (14.9)	38 (76.0)	35 (57.4)	4 (5.7)	<0.001	268	0
aortic valve and/or sinus repair	168 (62.7)	69 (79.3)	12 (24.0)	24 (39.3)	63 (90.0)		268	0
ascending aortic replacement	10 (3.7)	5 (5.7)	0 (0.0)	2 (3.3)	3 (4.3)		268	0
Mechanical valve: Yes	71 (26.5)	8 (9.2)	26 (52.0)	33 (54.1)	4 (5.7)	<0.001	268	0
Bioprosthetic valve: Yes	19 (7.1)	5 (5.7)	12 (24.0)	2 (3.3)	0 (0.0)	<0.001	268	0
Coronary artery bypass grafting: Yes	41 (15.3)	11 (12.6)	9 (18.0)	15 (24.6)	6 (8.6)	0.064	268	0
Operative exposure								
Operation duration, min	405.0 [358.8, 455.0]	395.0 [335.0, 445.0]	410.0 [366.2, 470.0]	435.0 [370.0, 470.0]	400.0 [370.1, 435.0]	0.095	268	0
Aortic cross-clamp time, min	144.4 (41.8)	133.8 (41.3)	153.9 (44.9)	156.5 (40.6)	140.3 (37.8)	0.004	268	0
Circulatory arrest time, min	19.3 (9.9)	17.9 (9.9)	19.5 (8.0)	21.6 (10.4)	18.9 (10.4)	0.205	263	5
Intraoperative transfusion								
Intraoperative red blood cell transfusion, U	5.92 ± 3.85	6.26 ± 3.75	6.50 ± 4.51	5.59 ± 3.34	5.37 ± 3.85	0.300	268	0
Intraoperative plasma, mL	600 [0, 800]	600 [0, 800]	600 [0, 800]	700 [600, 800]	800 [0, 800]	0.108	268	0
Intraoperative platelet transfusion, U	2.00 [1.00, 2.00]	2.00 [1.00, 2.00]	2.00 [1.00, 2.00]	2.00 [1.00, 2.00]	2.00 [1.00, 2.00]	0.120	268	0
Intraoperative cryoprecipitate transfusion, U	6.00 [6.00, 6.00]	6.00 [6.00, 6.00]	6.00 [6.00, 6.00]	6.00 [6.00, 6.00]	6.00 [6.00, 6.00]	0.530	268	0

Values are mean (standard deviation), median [25th, 75th percentiles], or n (%), as shown; percentages use the observed denominator. Operation, aortic cross-clamp, and circulatory-arrest durations and intraoperative plasma amount use global Kruskal–Wallis *P* values; primary-entry-tear location uses Pearson chi-square. Other rows use their variable-specific tests. Fisher exact Monte Carlo tests in this table used 100,000 replicates. PTA, prothrombin time activity; NT-proBNP, N-terminal pro-B-type natriuretic peptide; CABG, coronary artery bypass grafting. Transfusion quantities reported in U are presented in the units recorded in the clinical records.

Preoperative documentation of shock, tamponade, and organ-specific malperfusion by trajectory group is summarized descriptively in Supplementary Table S11.

### 3.4 Subsequent mortality after POD7

During follow-up after POD7, 27 deaths occurred among 268 trajectory participants. Deaths by group were 9/87 in the moderate-stable group, 11/50 in the low-declining group, 6/61 in the preserved-rapidly-declining group, and 1/70 in the high-preserved group. Kaplan–Meier cumulative mortality differed across groups (global log-rank *P* = 0.00210; Figure 3). The estimated cumulative mortality by POD365 was 10.5% (95% CI, 5.6%–19.3%), 24.0% (13.9%–39.4%), 10.0% (4.6%–20.8%), and 1.4% (0.2%–9.7%), respectively.

**Figure 3:**
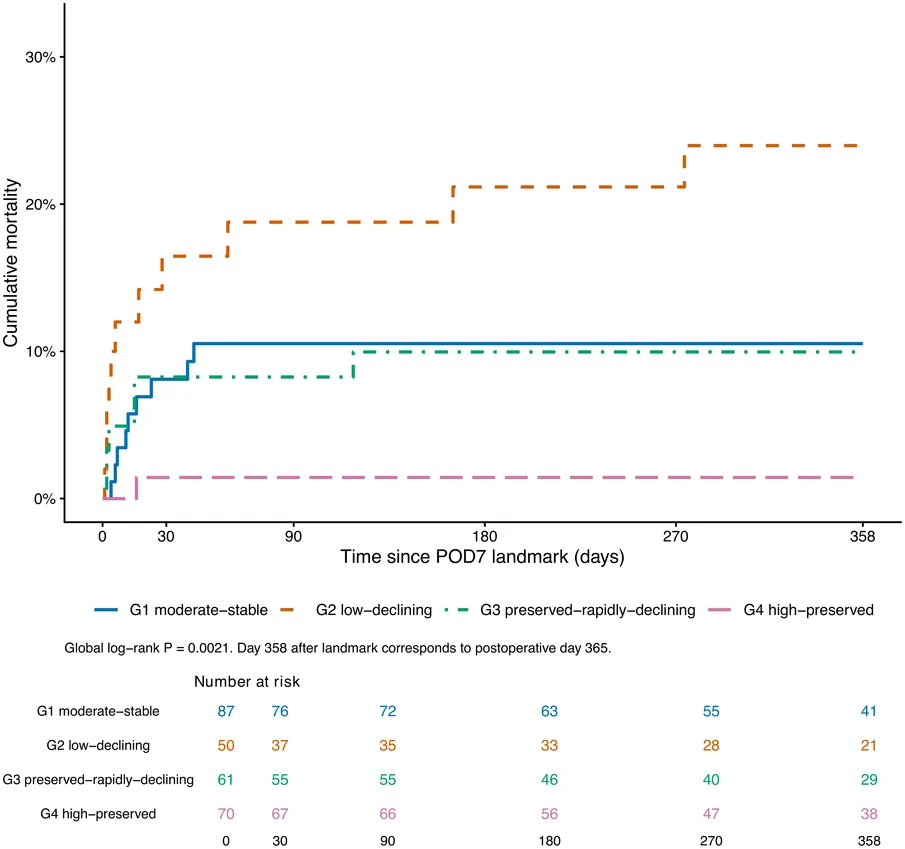
Landmark cumulative mortality by PTA trajectory group. Kaplan–Meier cumulative mortality curves begin immediately after POD7. Time is expressed as days since the POD7 landmark; day 358 corresponds to POD365. Numbers at risk are displayed beneath the plot at 0, 30, 90, 180, 270, and 358 days. The global log-rank *P* value was 0.00210. The high-preserved group had one death, so class-specific relative estimates are sparse. POD, postoperative day; PTA, prothrombin time activity.

Trajectory group remained globally associated with subsequent mortality in the age- and sex-adjusted Cox model (partial likelihood-ratio *P* = 0.0322) and the Firth sensitivity analysis (global *P* = 0.0481). Class-specific estimates were imprecise because only one death occurred in the high-preserved reference group. In the direct low-declining-versus-preserved-rapidly-declining comparison, the age- and sex-adjusted standard Cox hazard ratio was 1.543 (95% CI, 0.544–4.378; *P* = 0.4153), and the Firth hazard ratio was 1.503 (95% CI, 0.558–4.379; *P* = 0.4250). No clear difference in subsequent mortality was demonstrated between the low-declining and preserved-rapidly-declining trajectory groups (Table 2).

**Table 2:** Association between perioperative PTA trajectory group and all-cause mortality after POD7.

Trajectory group	n/deaths	KM cumulative mortality by POD365, % (95% CI)	Unadjusted HR (95% CI)	Age- and sex-adjusted HR (95% CI)	Firth-adjusted HR (95% CI)
Moderate-stable	87/9	10.5 (5.6–19.3)	7.60 (0.96–59.96)	5.56 (0.70–44.07)	3.91 (0.90–36.59)
Low-declining	50/11	24.0 (13.9–39.4)	17.99 (2.32–139.38)	10.54 (1.32–84.21)	7.42 (1.68–69.92)
Preserved-rapidly-declining	61/6	10.0 (4.6–20.8)	7.24 (0.87–60.11)	6.83 (0.82–56.81)	4.94 (1.04–47.39)
High-preserved	70/1	1.4 (0.2–9.7)	Reference	Reference	Reference

Analysis began immediately after POD7 and ended at death, last known follow-up, or POD365. Kaplan–Meier global log-rank *P* = 0.00210. Age- and sex-adjusted Cox global partial likelihood-ratio *P* = 0.0322; Firth global *P* = 0.0481. The high-preserved group had one death; class-specific estimates are therefore sparse and imprecise, and the global trajectory tests are the primary inferential results. The direct G2-versus-G3 contrast, specified before survival outcomes were examined, yielded an age- and sex-adjusted standard Cox HR of 1.543 (95% CI, 0.544–4.378; *P* = 0.4153) and a Firth HR of 1.503 (95% CI, 0.558–4.379; *P* = 0.4250). CI, confidence interval; HR, hazard ratio; KM, Kaplan–Meier; POD, postoperative day; PTA, prothrombin time activity.

### 3.5 Incremental information beyond the last preoperative PTA

The incremental analysis included 250 patients and 26 deaths. Adding the four-group trajectory variable to age, sex, and last preoperative PTA did not meet the prespecified likelihood-ratio criterion (M1 versus M3: likelihood-ratio statistic, 3.629; 3 df; *P* = 0.304; Firth penalized-likelihood *P* = 0.380). Relative to M1, the optimism-corrected M3 differences were 0.008 for Harrell C (95% bootstrap interval, −0.058 to 0.036), 0.008 for Uno C (−0.056 to 0.035), <0.001 for the day-358 AUC (−0.079 to 0.030), and 0.002 for the Brier score (−0.002 to 0.014, lower values indicate better performance). The bootstrap-corrected calibration slope was 0.934 for M1 and 0.664 for M3 (Table 3). Thus, this analysis did not show clear incremental information from K4 membership beyond last preoperative PTA, age, and sex.

**Table 3:** Incremental information from four-group PTA trajectory classification beyond last preoperative PTA.

Model	Predictors	n/deaths	Log likelihood	AIC	Harrell C	Uno C	AUC at day 358	Brier score	Calibration slope
M0	Age + sex	250/26	−129.174	262.347	0.731	0.740	0.759	0.087	0.962
M1	M0 + last preoperative PTA	250/26	−124.440	254.879	0.778	0.783	0.810	0.084	0.934
M2	M0 + K4 trajectory group	250/26	−125.495	260.990	0.759	0.768	0.783	0.089	0.642
M3	M0 + last preoperative PTA + K4 trajectory group	250/26	−122.625	257.250	0.786	0.791	0.810	0.086	0.664

Log likelihood and AIC are apparent model-fit statistics. Discrimination, Brier score, and calibration slope are optimism-corrected estimates from 500 bootstrap repetitions. The prespecified M1-versus-M3 likelihood-ratio statistic was 3.629 (3 df; *P* = 0.304), with *Δ*AIC =  + 2.371 for M3 relative to M1; the Firth penalized-likelihood *P* value was 0.380. Corrected M3-minus-M1 differences (95% bootstrap interval) were 0.008 (−0.058 to 0.036) for Harrell C, 0.008 (−0.056 to 0.035) for Uno C, <0.001 (−0.079 to 0.030) for AUC, and 0.002 (−0.002 to 0.014) for Brier score; lower Brier scores indicate better performance. AUC, area under the time-dependent receiver operating characteristic curve; PTA, prothrombin time activity.

### 3.6 Sensitivity and complementary analyses

A linear functional form was retained for last preoperative PTA (nonlinearity *P* = 0.698). In S1, POD1 PTA was associated with subsequent mortality (hazard ratio per 10 percentage points, 0.800; 95% CI, 0.656–0.974). In S2, the change from last preoperative PTA to POD1 had a hazard ratio per 10 percentage points of 0.927 (95% CI, 0.727–1.183). The primary S0-versus-S2 comparison did not show clear incremental information from the change term (likelihood-ratio statistic, 0.366; 1 df; *P* = 0.545). No POD3 or POD7 summary analysis was added *post hoc* (Supplementary Table S6).

The optimized K3 and K5 solutions preserved the broad contrast between declining and preserved patterns, and the posterior-probability ≥0.80 subset comprised 214 patients assigned as 66, 41, 47, and 60 to K4 groups 1–4. In that subset, deaths were 8, 10, 5, and 1 and the log-rank *P* value was 0.00392. No Cox model was fitted in this subset because the high-preserved reference group again contained only one death (Supplementary Tables S3–S5).

After outcome-blind exclusion of mechanical-valve recipients, trajectories were refitted in 197 patients with 20 deaths. Agreement with the primary K4 assignment was high (ARI, 0.851), with refitted group counts of 74, 28, 32, and 63. The post-landmark log-rank *P* value was 0.000748, the adjusted Cox global *P* value was 0.00287, and the Firth global *P* value was 0.00831. The broad longitudinal structure persisted, although the preserved-rapidly-declining trajectory was attenuated (Supplementary Table S7). Excluding any prosthetic-valve recipient left 178 patients; broad shapes were partly retained but membership shifted materially (ARI, 0.339). Consistent with the prespecified structural-only role of this analysis, survival outcomes were not examined (Supplementary Table S7).

The complementary flexible joint longitudinal-survival model and its simplified baseline-hazard rescue specifications did not satisfy the prespecified MCMC convergence criteria. No joint-model association estimate was retained for inference.

## 4 Discussion

In this cohort of adults undergoing ATAAD repair, outcome-blind modeling identified four reproducible perioperative PTA trajectory groups that differed in both level and shape: moderate-stable, low-declining, preserved-rapidly-declining, and high-preserved. Classification diagnostics, multistart agreement, bootstrap resampling, and a high-completeness analysis supported the four-group representation. Mortality after the POD7 landmark differed globally among trajectory groups, but the distinct morphologies of the low-declining and preserved-rapidly-declining groups did not translate into a clearly demonstrated adjusted mortality difference. Moreover, trajectory-group membership did not show clear incremental prognostic information beyond last preoperative PTA, age, and sex. Excluding mechanical-valve recipients and refitting the trajectories largely preserved the principal structure, although the rapid decline was attenuated. A complementary joint model did not meet predefined convergence criteria, so no association estimate from that model was reportable. Together, these findings support a trajectory-based description of perioperative PTA patterns rather than an ordered risk classification or prediction model.

The four trajectories provide a concise representation of heterogeneous perioperative PTA courses. The moderate-stable group began with moderately reduced PTA and remained comparatively stable, whereas the low-declining group entered surgery with lower PTA and declined further. The preserved-rapidly-declining group started at a relatively preserved level but showed a marked postoperative fall, and the high-preserved group maintained higher PTA throughout the observation window. Several processes could plausibly contribute to these patterns. ATAAD-related coagulation activation and consumption may already be present before repair, while cardiopulmonary bypass, hypothermia or circulatory arrest, operative injury, bleeding, transfusion, and hemostatic treatment can influence the postoperative course (4–7, 18–22). Differences in systemic illness or hepatic and metabolic function could also contribute, but the present data do not establish those pathways. The observed differences in cross-clamp duration, proximal procedure, and prosthetic-valve status place these PTA trajectories within a broader perioperative course and may contribute to their longitudinal structure. The trajectories summarize the net longitudinal PTA pattern observed under the combined influence of baseline disease state, anatomy, operative exposure, bleeding and transfusion, and postoperative treatment. They are therefore best understood as composite perioperative patterns rather than as discrete readouts of intrinsic coagulation biology; the relative contributions of disease and treatment to each pattern remain unresolved. The outcome-blind selection process is important in this respect: the trajectory groups were selected because they offered a stable, parsimonious representation of longitudinal PTA, not because they maximized separation of survival curves (11–13). Nor should the groups be assumed to be discrete biological entities. Like other latent-class summaries, they approximate a distribution of individual longitudinal courses, and posterior assignment carries classification uncertainty. The high average posterior probabilities and sensitivity analyses reduce, but do not remove, that uncertainty.

The contrast between the low-declining and preserved-rapidly-declining trajectory groups is especially informative. Both reached low PTA levels during the postoperative window, but they arrived there through different paths: one from an already reduced preoperative level and the other after a pronounced postoperative decline. This distinction illustrates how a longitudinal trajectory can retain information about temporal morphology that a single postoperative measurement cannot fully represent. It does not, however, establish different mortality tiers. The direct age- and sex-adjusted comparison, specified after the trajectory solution was selected and before the survival outcomes were examined, did not demonstrate a clear difference; the Firth analysis led to the same interpretation. More broadly, the global post-landmark association is more dependable than the large class-specific hazard ratios because the high-preserved reference group had only one death. The landmark design preserves temporal ordering by beginning survival follow-up only after the PTA observation window (14, 15), but it also defines a conditional estimand: the results apply to patients who survived and remained observable through POD7 and had sufficient PTA data for trajectory-group assignment. They do not describe early postoperative mortality or support causal effects of trajectory-group membership.

The incremental analysis provides an equally important boundary on interpretation. Adding the four-group trajectory classification to age, sex, and last preoperative PTA did not meet the prespecified likelihood-ratio criterion, and the optimism-corrected performance differences were small and uncertain. Last preoperative PTA therefore already contained prognostic information that was not clearly augmented by K4 membership in this sample. This negative finding is not a failure of longitudinal description: the descriptive value of trajectory grouping and its incremental predictive value are different questions. The trajectories may summarize heterogeneous perioperative paths and generate hypotheses about disease and treatment context without improving discrimination in a sparse-event cohort. The simple PTA analyses reinforce this distinction. POD1 PTA was associated with subsequent mortality, whereas the preoperative-to-POD1 change did not show clear incremental information beyond preoperative PTA. Thus, the results do not support a general claim that an arbitrary early change score or a trajectory label is a superior prognostic measure. They instead suggest that the appropriate role of trajectory grouping in this dataset is descriptive and hypothesis-generating. Future work should compare prespecified longitudinal summaries only after confirming their measurement reliability and should separate evaluation of morphology, association, and incremental prediction rather than treating them as interchangeable endpoints.

Mechanical-valve implantation was more frequent in the declining trajectory groups. Because mechanical-valve replacement entails a distinct postoperative antithrombotic context (23), we examined the stability of the trajectory structure after excluding these patients. The broad four-pattern structure persisted, although the decline in the preserved-rapidly-declining trajectory was attenuated. This finding indicates that the overall structure was not confined to mechanical-valve recipients, but it does not establish independence from postoperative antithrombotic treatment because patient-level treatment exposure, including exposure among patients without mechanical valves, was unavailable.

The observation process also warrants explicit caution. PTA was obtained during routine care rather than on a research-mandated schedule, and measurement frequency varied with early death, continuous renal replacement therapy, delayed sternal closure, and the intensity and duration of ICU or hospital care. The visit process may therefore have been informative: sicker or more intensively managed patients could contribute measurements at different frequencies or times. GBTM used the observed measurements without interpolation, last observation carried forward, or imputation, which avoids creating unobserved PTA values but does not eliminate selection related to when measurements were obtained. Joint longitudinal-survival models can accommodate irregular measurements and death-related dropout under their assumptions, but standard formulations do not automatically model an informative visit-intensity process. Here, neither the primary flexible joint model nor prespecified simplified baseline-hazard specifications satisfied the predefined MCMC convergence criteria (17). No posterior association estimate was therefore reported, and the analysis cannot support inference about a continuously updated latent PTA process and mortality. Nonconvergence should not be interpreted as evidence of no association.

Strengths of the study include an explicitly aligned POD7 landmark, outcome-blind trajectory selection, multiple classification and stability checks, a prespecified comparison of the two declining morphologies, and transparent evaluation of incremental information. Limitations include the single-center retrospective design, the modest cohort, and 27 post-landmark deaths. The POD7 landmark excludes early deaths and restricts inference to patients who survived and remained observable through POD7 with sufficient PTA measurements. PTA was measured during routine care on an irregular schedule, and the observation process may therefore have been informative. Important determinants of perioperative PTA could not be fully resolved in the retrospective data. Preoperative shock, tamponade, and malperfusion were incompletely ascertained, and some anatomical and operative characteristics, including CPB duration and cohort-wide false-lumen status, were unavailable or incomplete; residual confounding by baseline disease severity therefore cannot be excluded. In addition, plasma administration and postoperative anticoagulation could not be aligned reliably with individual PTA measurements. Because these treatments may both respond to the evolving clinical state and influence subsequent PTA, disease-related, procedural, and treatment-related contributions to the observed trajectories cannot be separated reliably. Given the limited number of post-landmark deaths, multivariable adjustment was deliberately parsimonious. External reproducibility remains to be established.

The clinical implications are therefore deliberately modest. Characterizing perioperative PTA trajectories may provide a useful way to conceptualize heterogeneous longitudinal PTA patterns and to structure postoperative reassessment or research stratification. The different paths of the two declining trajectories may generate hypotheses about distinct combinations of preoperative disease burden, operative exposure, and treatment. Trajectory-group assignment, however, requires observations through POD7 and is unavailable at surgery; the study did not evaluate a real-time classifier, treatment guidance, or PTA-guided anticoagulation. Prospective multicenter studies with standardized sampling, recoverable assay metadata, time-aligned treatment data, and sufficient events are needed to test external reproducibility, clarify the observation process, and determine whether longitudinal PTA morphology has value beyond careful interpretation of individual measurements. Such studies should enroll across centers and assay platforms, prespecify the timing of PTA and treatment capture, retain early deaths in a parallel estimand, and distinguish structural replication of the trajectory patterns from any later evaluation of clinical utility.

## 5 Conclusion

Four reproducible perioperative PTA trajectories characterized distinct longitudinal patterns from the last preoperative measurement through POD7 and were associated with subsequent all-cause mortality after a POD7 landmark. These trajectories are best interpreted as composite perioperative patterns reflecting both disease and treatment context rather than as intrinsic coagulation states. The observed association with mortality should not be interpreted as evidence of an independent biological mechanism linking the trajectories to mortality. They were not ordered mortality-risk tiers and did not show clear incremental prognostic information beyond last preoperative PTA. External validation with standardized sampling and time-aligned treatment data is required before clinical implementation is considered.

## Data Availability

The datasets presented in this article are not readily available because the dataset contains patient-level clinical information and is subject to privacy and ethical restrictions. It is therefore not publicly available or routinely shareable. Requests for access may be considered on reasonable request to the corresponding author, subject to institutional approval and applicable data-protection requirements. Requests to access the datasets should be directed to Weiping Li, wpli818@126.com.
