# Supplementary material for: Perioperative prothrombin time activity trajectories and subsequent mortality after surgical repair of acute type A aortic dissection
Source: Front Med (Lausanne). 2026 Oct 19;13:1885240. doi: 10.3389/fmed.2026.1885240 (PMC13724389; doi:10.3389/fmed.2026.1885240)
Supplement: Supplementary file 1 [file Supplementaryfile1.docx]

Supplementary Material

**Perioperative Prothrombin Time Activity Trajectories and Subsequent Mortality After Surgical Repair of Acute Type A Aortic Dissection**

Supplementary Tables S1–S11.

# Supplementary Table S1. PTA availability at the last preoperative time point and POD1–7, with observed and missing denominators

## Panel A. Time-point availability among 269 patients observable at the POD7 landmark

| **Time point** | **Available, n (%)** | **Missing, n (%)** |
| --- | --- | --- |
| Preoperative | 250 (92.9) | 19 (7.1) |
| POD1 | 268 (99.6) | 1 (0.4) |
| POD2 | 259 (96.3) | 10 (3.7) |
| POD3 | 255 (94.8) | 14 (5.2) |
| POD4 | 238 (88.5) | 31 (11.5) |
| POD5 | 223 (82.9) | 46 (17.1) |
| POD6 | 207 (77.0) | 62 (23.0) |
| POD7 | 196 (72.9) | 73 (27.1) |

## Panel B. Distribution of observed trajectory time points

| **Observed time points (of 8)** | **Patients, n (%)** |
| --- | --- |
| 8 | 136 (50.6) |
| 7 | 61 (22.7) |
| 6 | 38 (14.1) |
| 5 | 22 (8.2) |
| 4 | 9 (3.3) |
| 3 | 2 (0.7) |
| 2 | 0 (0.0) |
| 1 | 1 (0.4) |
| 0 | 0 (0.0) |

*Complete eight-point observations were available for 136 patients; 133 had at least one unobserved time point. One patient had fewer than three valid observed PTA measurements and did not enter trajectory modeling.*

# Supplementary Table S2. Outcome-blind GBTM candidate models K2–K5 and selection diagnostics

| **K** | **Log likelihood** | **AIC** | **BIC** | **Valid starts / total** | **Best optimum recovered, %** | **Entropy** | **Distinct local maxima** |
| --- | --- | --- | --- | --- | --- | --- | --- |
| 2 | -8367.082 | 16756.2 | 16795.7 | 20/20 | 100.0 | 0.865 | No |
| 3 | -8277.639 | 16589.3 | 16650.3 | 50/50 | 98.0 | 0.828 | Yes |
| 4 | -8209.615 | 16465.2 | 16547.8 | 20/20 | 100.0 | 0.831 | No |
| 5 | -8171.356 | 16400.7 | 16504.9 | 50/50 | 98.0 | 0.846 | Yes |

*Models used 268 patients and 1,895 observed PTA measurements with group-specific residual standard deviations. AIC and BIC use the identifiable free-parameter count. Candidate-model diagnostics were assessed without survival outcomes. Supplementary Table S2 summarizes the initial all-cubic candidate-model screening fits, whereas Supplementary Table S3 reports the polynomial-order-optimized K3–K5 specifications used for the final structural comparison.*

# Supplementary Table S3. Optimized K3, K4, and K5 specifications, posterior classification, and structural comparison

## Panel A. Optimized model comparison

| **K** | **Polynomial orders** | **Valid starts / total** | **AIC** | **BIC** | **Entropy** | **Smallest class, %** | **Minimum AvePP** | **Minimum OCC** | **Minimum PP ≥0.80, %** |
| --- | --- | --- | --- | --- | --- | --- | --- | --- | --- |
| 3 | 3;2;2 | 30/30 | 16586.0 | 16639.9 | 0.826 | 27.2 | 0.893 | 17.32 | 77.6 |
| 4 | 2;3;3;2 | 30/30 | 16462.3 | 16537.7 | 0.830 | 18.7 | 0.897 | 18.33 | 75.9 |
| 5 | 3;0;2;3;2 | 50/50 | 16394.8 | 16481.0 | 0.826 | 12.3 | 0.868 | 19.88 | 71.0 |

## Panel B. K4 posterior classification

| **Group** | **Trajectory group** | **Assigned n (%)** | **Estimated proportion, %** | **AvePP** | **OCC** | **PP ≥0.80, %** |
| --- | --- | --- | --- | --- | --- | --- |
| G1 | Moderate-stable | 87 (32.5) | 32.1 | 0.897 | 18.33 | 75.9 |
| G2 | Low-declining | 50 (18.7) | 18.5 | 0.913 | 46.53 | 82.0 |
| G3 | Preserved-rapidly-declining | 61 (22.8) | 23.4 | 0.898 | 28.92 | 77.0 |
| G4 | High-preserved | 70 (26.1) | 26.0 | 0.922 | 33.89 | 85.7 |

*Normalized entropy for K4 was 0.830. AvePP, average posterior probability; OCC, odds of correct classification; PP, assigned posterior probability.*

# Supplementary Table S4. Bootstrap stability, high-completeness sensitivity, and posterior-probability sensitivity

## Panel A. Patient-level bootstrap stability

| **K** | **Target replicates** | **Successful fits** | **Successfully matched** | **Collapse/merge events** | **Unstable solutions** |
| --- | --- | --- | --- | --- | --- |
| 3 | 200 | 200 | 200 | 0 | 2 |
| 4 | 200 | 200 | 200 | 0 | 5 |
| 5 | 200 | 200 | 200 | 3 | 23 |

## Panel B. High-completeness sensitivity (at least 6 of 8 occasions observed)

| **K** | **n** | **Observed PTA values** | **Valid starts / total** | **Maximum class RMSE** | **ARI** | **NMI** | **Exact agreement, %** |
| --- | --- | --- | --- | --- | --- | --- | --- |
| 3 | 235 | 1743 | 20/20 | 0.79 | 0.975 | 0.958 | 99.1 |
| 4 | 235 | 1743 | 20/20 | 1.10 | 0.967 | 0.953 | 98.7 |
| 5 | 235 | 1743 | 50/50 | 1.08 | 0.989 | 0.987 | 99.6 |

*RMSE, root mean square error; ARI, adjusted Rand index; NMI, normalized mutual information.*

## Panel C. Assigned posterior probability ≥0.80 subset

| **Group** | **Trajectory group** | **n** | **Deaths** | **Censored** |
| --- | --- | --- | --- | --- |
| G1 | moderate-stable | 66 | 8 | 58 |
| G2 | low-declining | 41 | 10 | 31 |
| G3 | preserved-rapidly-declining | 47 | 5 | 42 |
| G4 | high-preserved | 60 | 1 | 59 |

*Subset n = 214; global log-rank P = 0.004. A Cox model was not fitted because G4 had one death.*

# Supplementary Table S5. Alternative K3/K5 survival descriptions and posterior-probability ≥0.80 subset

| **K** | **Group event counts** | **Global log-rank P** | **Age/sex-adjusted global P** | **Firth global P** | **Sparse-event note** |
| --- | --- | --- | --- | --- | --- |
| 3 | G1 = 17; G2 = 9; G3 = 1 | 0.008 | 0.013 | 0.024 | Yes: G3=1 |
| 4 | G1 = 9; G2 = 11; G3 = 6; G4 = 1 | 0.002 | 0.032 | 0.048 | Yes: G4=1 |
| 5 | G1 = 9; G2 = 8; G3 = 2; G4 = 7; G5 = 1 | 0.009 | 0.099 | 0.150 | Yes: G3=2; G5=1 |

*K3 and K5 are sensitivity descriptions and do not replace the primary K4 solution. The posterior-probability ≥0.80 K4 subset is reported in Supplementary Table S4.*

# Supplementary Table S6. Incremental models, simple PTA summaries, and proportional-hazards diagnostics

## Panel A. Standard Cox nested comparisons

| **Reduced** | **Extended** | **Question** | **LR** | **df** | **P** | **ΔAIC** |
| --- | --- | --- | --- | --- | --- | --- |
| M0 | M1 | preop PTA beyond age/sex | 9.468 | 1 | 0.002 | -7.468 |
| M0 | M2 | trajectory beyond age/sex | 7.358 | 3 | 0.061 | -1.358 |
| M1 | M3 | trajectory beyond preop PTA + age/sex | 3.629 | 3 | 0.304 | 2.371 |
| M2 | M3 | preop PTA beyond trajectory + age/sex | 5.739 | 1 | 0.017 | -3.739 |

*The prespecified primary comparison was M1 versus M3. M0, age and sex; M1, M0 plus last preoperative PTA; M2, M0 plus K4; M3, M0 plus last preoperative PTA and K4.*

## Panel B. Firth penalized-likelihood comparisons

| **Comparison** | **Penalized LR** | **df** | **P** | **Converged** |
| --- | --- | --- | --- | --- |
| F0 vs F1 | 9.388 | 1 | 0.002 | Yes |
| F0 vs F2 | 6.423 | 3 | 0.093 | Yes |
| F1 vs F3 | 3.076 | 3 | 0.380 | Yes |
| F2 vs F3 | 5.736 | 1 | 0.017 | Yes |

## Panel C. Primary paired M3-minus-M1 performance contrasts

| **Metric** | **Optimism-corrected difference** | **95% bootstrap interval** | **Valid paired replicates** |
| --- | --- | --- | --- |
| Harrell_C | 0.008 | -0.058 to 0.036 | 500 |
| Uno_C at day 358 | 0.008 | -0.056 to 0.035 | 500 |
| AUC at day 358 | <0.001 | -0.079 to 0.030 | 500 |
| Brier at day 358 | 0.002 | -0.002 to 0.014 | 500 |

*Positive differences favor M3 for C indices and AUC; negative differences favor M3 for Brier score.*

## Panel D. Simple PTA models

| **Model** | **PTA term (per 10 percentage points)** | **HR** | **95% CI** | **P** |
| --- | --- | --- | --- | --- |
| S0 | Last preoperative PTA | 0.734 | 0.603–0.894 | 0.002 |
| S1 | POD1 PTA | 0.800 | 0.656–0.974 | 0.027 |
| S2 | Last preoperative PTA | 0.709 | 0.565–0.890 | 0.003 |
| S2 | Preoperative-to-POD1 change | 0.927 | 0.727–1.183 | 0.543 |

*S0 included age, sex, and last preoperative PTA; S1 included age, sex, and POD1 PTA; S2 included age, sex, last preoperative PTA, and preoperative-to-POD1 change. S0 versus S2: LR = 0.366; P = .545.*

## Panel E. Selected proportional-hazards diagnostics

| **Model** | **Term** | **χ²** | **df** | **P** |
| --- | --- | --- | --- | --- |
| M1 | age | 5.569 | 1 | 0.018 |
| M3 | age | 5.275 | 1 | 0.022 |
| M3 | K4 group 1 | 1.255 | 1 | 0.263 |
| M3 | K4 group 2 | 0.000 | 1 | 0.993 |
| M3 | K4 group 3 | 2.444 | 1 | 0.118 |

*Diagnostics used the Kaplan–Meier time transform. The time-varying-age M1-versus-M3 comparison yielded LR = 3.382 (3 df; P = .336).*

# Supplementary Table S7. Mechanical-valve-exclusion and any-prosthetic-valve-exclusion structural analyses

## Panel A. Outcome summary

| **Cohort** | **n** | **Deaths** | **Global log-rank P** | **Age/sex-adjusted global P** | **Firth global P** |
| --- | --- | --- | --- | --- | --- |
| Primary K4 | 268 | 27 | 0.002 | 0.032 | 0.048 |
| Mechanical-valve-excluded refit | 197 | 20 | <0.001 | 0.003 | 0.008 |

*Mechanical-valve exclusion was used to examine trajectory stability in patients without mechanical-valve implantation.*

## Panel B. Class distributions in the mechanical-valve-excluded cohort

| **Assignment** | **Group** | **n** | **%** |
| --- | --- | --- | --- |
| Primary assignment within restricted cohort | G1 | 79 | 40.1 |
| Primary assignment within restricted cohort | G2 | 24 | 12.2 |
| Primary assignment within restricted cohort | G3 | 28 | 14.2 |
| Primary assignment within restricted cohort | G4 | 66 | 33.5 |
| Restricted-cohort refit | G1 | 74 | 37.6 |
| Restricted-cohort refit | G2 | 28 | 14.2 |
| Restricted-cohort refit | G3 | 32 | 16.2 |
| Restricted-cohort refit | G4 | 63 | 32.0 |

## Panel C. Structural agreement

| **Metric** | **Value** |
| --- | --- |
| Exact assignment agreement | 0.934 |
| Adjusted Rand index | 0.851 |
| Normalized mutual information | 0.817 |

*The refitted G3 decline was attenuated. The any-prosthetic-valve-exclusion analysis retained a stable four-class fit but did not reproduce all primary shape features and was therefore interpreted structurally only.*

# Supplementary Table S8. Expanded perioperative and postoperative descriptive characteristics

| **Variable** | **Overall** | **G1** | **G2** | **G3** | **G4** | **Global P** | **Available n** | **Missing n** |
| --- | --- | --- | --- | --- | --- | --- | --- | --- |
| Intraoperative red blood cells, U | 5.92 ± 3.85 | 6.26 ± 3.75 | 6.50 ± 4.51 | 5.59 ± 3.34 | 5.37 ± 3.85 | 0.300 | 268 | 0 |
| Intraoperative platelets, U | 2.00 [1.00, 2.00] | 2.00 [1.00, 2.00] | 2.00 [1.00, 2.00] | 2.00 [1.00, 2.00] | 2.00 [1.00, 2.00] | 0.120 | 268 | 0 |
| Intraoperative cryoprecipitate, U | 6.00 [6.00, 6.00] | 6.00 [6.00, 6.00] | 6.00 [6.00, 6.00] | 6.00 [6.00, 6.00] | 6.00 [6.00, 6.00] | 0.530 | 268 | 0 |
| Atrial fibrillation: Yes | 15 (5.6) | 9 (10.3) | 1 (2.0) | 2 (3.3) | 3 (4.3) | 0.172 | 268 | 0 |
| Delayed sternal closure: Yes | 24 (9.0) | 7 (8.0) | 8 (16.0) | 3 (4.9) | 6 (8.6) | 0.254 | 268 | 0 |
| Continuous renal replacement therapy: Yes | 47 (17.5) | 14 (16.1) | 14 (28.0) | 8 (13.1) | 11 (15.7) | 0.180 | 268 | 0 |
| Postoperative renal dysfunction: Yes | 33 (12.3) | 12 (13.8) | 9 (18.0) | 6 (9.8) | 6 (8.6) | 0.403 | 268 | 0 |
| Mechanical ventilation duration, days | 2.00 [0.00, 4.25] | 2.00 [0.00, 6.00] | 2.00 [0.00, 4.75] | 2.00 [1.00, 4.00] | 2.00 [1.00, 4.00] | 0.928 | 268 | 0 |
| ICU stay, h | 163.82 [98.67, 318.72] | 158.38 [81.78, 375.88] | 176.54 [117.93, 365.50] | 196.95 [124.12, 289.97] | 146.78 [90.44, 269.40] | 0.483 | 268 | 0 |
| Hospital stay, days | 20.55 [16.21, 25.45] | 20.87 [16.92, 25.98] | 20.15 [15.15, 25.57] | 20.48 [15.52, 25.42] | 20.38 [16.84, 23.92] | 0.624 | 268 | 0 |

*Values are mean ± standard deviation, median [interquartile range], or n (%). Global comparisons used the variable-specific tests described in the Methods. Transfusion quantities reported in U are presented in the units recorded in the clinical records.*

# Supplementary Table S9. Follow-up, death timing, and censoring completeness

## Panel A. Follow-up summary

| **Measure** | **Value** |
| --- | --- |
| Final study cohort | 284 |
| Deaths through POD365 | 42 |
| Right-censored observations | 242 |
| Non-decedents censored before POD365 | 113 |
| Non-decedents censored at POD365 | 129 |
| Observed follow-up, median [Q1, Q3], days | 333.5 [150.5, 365.0] |
| Observed follow-up range, days | 2–365 |

## Panel B. Death timing

| **Interval** | **Deaths, n** |
| --- | --- |
| POD0–7 | 14 |
| POD8–30 | 20 |
| POD31–90 | 5 |
| POD91–180 | 2 |
| POD181–365 | 1 |

## Panel C. In-hospital and post-discharge deaths

| **Death location** | **Deaths, n** |
| --- | --- |
| In hospital | 31 |
| After discharge | 11 |

*Cause-specific death was not reported because causes were not systematically available for reliable adjudication. Right-censoring before POD365 was retained; complete one-year follow-up was not assumed.*

**Supplementary Table S10. Operative, anatomical, proximal-procedure, and plasma context by PTA trajectory group.**

**Panel A. Operative exposure and arch procedure**

| **Variable / level** | **Overall** | **G1** | **G2** | **G3** | **G4** | **Observed n** | **Global P** |
| --- | --- | --- | --- | --- | --- | --- | --- |
| Aortic cross-clamp duration (min) — mean (SD) | 144.4 (41.8) | 133.8 (41.3) | 153.9 (44.9) | 156.5 (40.6) | 140.3 (37.8) | 268 | 0.004 |
| Aortic cross-clamp duration (min) — median [Q1, Q3] | 145.2 [115.8, 175.0] | 130.0 [100.5, 161.5] | 149.0 [129.0, 178.8] | 161.0 [127.0, 189.0] | 140.0 [116.0, 165.6] | 268 |  |
| Aortic cross-clamp duration (min) — n observed | 268 | 87 | 50 | 61 | 70 | 268 |  |
| Circulatory-arrest duration (min) — mean (SD) | 19.3 (9.9) | 17.9 (9.9) | 19.5 (8.0) | 21.6 (10.4) | 18.9 (10.4) | 263 | 0.205 |
| Circulatory-arrest duration (min) — median [Q1, Q3] | 20.0 [11.0, 27.0] | 16.0 [10.0, 27.0] | 21.0 [12.0, 25.0] | 23.0 [13.0, 28.0] | 20.0 [10.0, 28.0] | 263 |  |
| Circulatory-arrest duration (min) — n observed | 263 | 85 | 49 | 61 | 68 | 263 |  |
| Total operation duration (min) — mean (SD) | 413.7 (87.7) | 399.8 (89.0) | 438.7 (110.8) | 417.5 (69.6) | 409.7 (79.0) | 268 | 0.095 |
| Total operation duration (min) — median [Q1, Q3] | 405.0 [358.8, 455.0] | 395.0 [335.0, 445.0] | 410.0 [366.2, 470.0] | 435.0 [370.0, 470.0] | 400.0 [370.1, 435.0] | 268 |  |
| Total operation duration (min) — n observed | 268 | 87 | 50 | 61 | 70 | 268 |  |
| Circulatory-arrest temperature (degrees C) — mean (SD) | 27.1 (1.6) | 27.4 (1.4) | 27.1 (1.5) | 26.9 (1.7) | 27.0 (1.7) | 240 | 0.130 |
| Circulatory-arrest temperature (degrees C) — median [Q1, Q3] | 28.0 [25.0, 28.0] | 28.0 [28.0, 28.0] | 28.0 [25.0, 28.0] | 28.0 [25.0, 28.0] | 28.0 [25.0, 28.0] | 240 |  |
| Circulatory-arrest temperature (degrees C) — n observed | 240 | 79 | 45 | 57 | 59 | 240 |  |
| Arch procedure — no arch replacement | 1/267 (0.4%) | 0/87 (0.0%) | 0/50 (0.0%) | 0/60 (0.0%) | 1/70 (1.4%) | 267 | 0.255 |
| Arch procedure — HAR / partial arch | 23/267 (8.6%) | 5/87 (5.7%) | 7/50 (14.0%) | 7/60 (11.7%) | 4/70 (5.7%) | 267 |  |
| Arch procedure — TAR / total arch | 243/267 (91.0%) | 82/87 (94.3%) | 43/50 (86.0%) | 53/60 (88.3%) | 65/70 (92.9%) | 267 |  |
| Frozen elephant trunk / elephant-trunk stent recorded | 240/268 (89.6%) | 80/87 (92.0%) | 43/50 (86.0%) | 53/61 (86.9%) | 64/70 (91.4%) | 268 | 0.585 |

**Panel B. Recorded anatomy**

| **Variable / level** | **Overall** | **G1** | **G2** | **G3** | **G4** | **Observed n** | **Global P** |
| --- | --- | --- | --- | --- | --- | --- | --- |
| Primary entry tear location — Ascending aorta | 115/263 (43.7%) | 27/84 (32.1%) | 33/50 (66.0%) | 35/61 (57.4%) | 20/68 (29.4%) | 263 | <0.001 |
| Primary entry tear location — Aortic arch/arch branches | 70/263 (26.6%) | 30/84 (35.7%) | 9/50 (18.0%) | 13/61 (21.3%) | 18/68 (26.5%) | 263 |  |
| Primary entry tear location — Descending aorta | 36/263 (13.7%) | 13/84 (15.5%) | 4/50 (8.0%) | 3/61 (4.9%) | 16/68 (23.5%) | 263 |  |
| Primary entry tear location — No definite primary entry identified | 42/263 (16.0%) | 14/84 (16.7%) | 4/50 (8.0%) | 10/61 (16.4%) | 14/68 (20.6%) | 263 |  |
| Arch involvement — Recorded involvement / finding | 107/109 (98.2%) | 34/35 (97.1%) | 24/24 (100.0%) | 22/23 (95.7%) | 27/27 (100.0%) | 109 | 0.696 |
| Descending thoracic involvement — Recorded involvement / finding | 98/109 (89.9%) | 31/35 (88.6%) | 21/24 (87.5%) | 21/23 (91.3%) | 25/27 (92.6%) | 109 | 0.946 |
| Abdominal aortic involvement — Recorded involvement / finding | 94/109 (86.2%) | 30/35 (85.7%) | 21/24 (87.5%) | 20/23 (87.0%) | 23/27 (85.2%) | 109 | 1.000 |
| Brachiocephalic involvement — Recorded involvement / finding | 61/109 (56.0%) | 17/35 (48.6%) | 16/24 (66.7%) | 12/23 (52.2%) | 16/27 (59.3%) | 109 | 0.543 |
| Left common carotid involvement — Recorded involvement / finding | 46/109 (42.2%) | 14/35 (40.0%) | 12/24 (50.0%) | 9/23 (39.1%) | 11/27 (40.7%) | 109 | 0.854 |
| Left subclavian involvement — Recorded involvement / finding | 51/109 (46.8%) | 13/35 (37.1%) | 13/24 (54.2%) | 12/23 (52.2%) | 13/27 (48.1%) | 109 | 0.548 |
| Celiac trunk involvement — Recorded involvement / finding | 57/109 (52.3%) | 15/35 (42.9%) | 16/24 (66.7%) | 12/23 (52.2%) | 14/27 (51.9%) | 109 | 0.356 |
| Iliac arterial thrombosis — Recorded involvement / finding | 3/109 (2.8%) | 0/35 (0.0%) | 2/24 (8.3%) | 0/23 (0.0%) | 1/27 (3.7%) | 109 | 0.190 |
| Renal artery origin from false lumen — No recorded renal artery origin from false lumen | 38/109 (34.9%) | 14/35 (40.0%) | 7/24 (29.2%) | 8/23 (34.8%) | 9/27 (33.3%) | 109 | 0.971 |
| Renal artery origin from false lumen — Left | 38/109 (34.9%) | 11/35 (31.4%) | 10/24 (41.7%) | 8/23 (34.8%) | 9/27 (33.3%) | 109 |  |
| Renal artery origin from false lumen — Right | 22/109 (20.2%) | 6/35 (17.1%) | 6/24 (25.0%) | 5/23 (21.7%) | 5/27 (18.5%) | 109 |  |
| Renal artery origin from false lumen — Bilateral | 11/109 (10.1%) | 4/35 (11.4%) | 1/24 (4.2%) | 2/23 (8.7%) | 4/27 (14.8%) | 109 |  |

**Panel C. Proximal procedure, aortic regurgitation, and prosthetic valve**

| **Variable / level** | **Overall** | **G1** | **G2** | **G3** | **G4** | **Observed n** | **Global P** |
| --- | --- | --- | --- | --- | --- | --- | --- |
| Proximal aortic procedure — Aortic valve and/or sinus repair | 168/268 (62.7%) | 69/87 (79.3%) | 12/50 (24.0%) | 24/61 (39.3%) | 63/70 (90.0%) | 268 | <0.001 |
| Proximal aortic procedure — Bentall | 90/268 (33.6%) | 13/87 (14.9%) | 38/50 (76.0%) | 35/61 (57.4%) | 4/70 (5.7%) | 268 |  |
| Proximal aortic procedure — Ascending-aortic replacement only / other recorded category | 10/268 (3.7%) | 5/87 (5.7%) | 0/50 (0.0%) | 2/61 (3.3%) | 3/70 (4.3%) | 268 |  |
| Prosthetic aortic valve type — No prosthetic valve recorded | 178/268 (66.4%) | 74/87 (85.1%) | 12/50 (24.0%) | 26/61 (42.6%) | 66/70 (94.3%) | 268 | <0.001 |
| Prosthetic aortic valve type — Mechanical valve | 71/268 (26.5%) | 8/87 (9.2%) | 26/50 (52.0%) | 33/61 (54.1%) | 4/70 (5.7%) | 268 |  |
| Prosthetic aortic valve type — Bioprosthetic valve | 19/268 (7.1%) | 5/87 (5.7%) | 12/50 (24.0%) | 2/61 (3.3%) | 0/70 (0.0%) | 268 |  |
| Aortic regurgitation grade — Not obvious or not documented | 24/268 (9.0%) | 12/87 (13.8%) | 4/50 (8.0%) | 2/61 (3.3%) | 6/70 (8.6%) | 268 | <0.001 |
| Aortic regurgitation grade — Mild | 117/268 (43.7%) | 46/87 (52.9%) | 11/50 (22.0%) | 20/61 (32.8%) | 40/70 (57.1%) | 268 |  |
| Aortic regurgitation grade — Moderate | 58/268 (21.6%) | 15/87 (17.2%) | 13/50 (26.0%) | 13/61 (21.3%) | 17/70 (24.3%) | 268 |  |
| Aortic regurgitation grade — Severe | 69/268 (25.7%) | 14/87 (16.1%) | 22/50 (44.0%) | 26/61 (42.6%) | 7/70 (10.0%) | 268 |  |
| Cross-clamp duration (min) — Aortic valve and/or sinus repair | 131.0 [102.0, 155.8]; n=168 | 125.0 [99.0, 155.6]; n=69 | 127.0 [95.5, 143.7]; n=12 | 125.5 [105.8, 149.5]; n=24 | 141.0 [116.0, 163.8]; n=63 | 168 |  |
| Cross-clamp duration (min) — Bentall | 174.5 [147.8, 193.0]; n=90 | 182.0 [158.0, 206.0]; n=13 | 160.2 [136.5, 180.0]; n=38 | 183.0 [162.5, 195.5]; n=35 | 180.0 [155.5, 198.5]; n=4 | 90 |  |
| Cross-clamp duration (min) — Ascending-aortic replacement only / other recorded category | 89.5 [74.0, 105.5]; n=10 | 94.0 [85.0, 104.0]; n=5 | NA; n=0 | 98.5 [82.2, 114.8]; n=2 | 74.0 [60.0, 102.0]; n=3 | 10 |  |
| Circulatory-arrest duration (min) — Aortic valve and/or sinus repair | 16.0 [10.0, 27.0]; n=165 | 15.0 [10.0, 27.0]; n=67 | 16.5 [10.0, 25.5]; n=12 | 16.5 [9.8, 28.2]; n=24 | 19.0 [10.0, 27.8]; n=62 | 165 |  |
| Circulatory-arrest duration (min) — Bentall | 23.0 [17.0, 27.8]; n=89 | 22.0 [18.0, 29.0]; n=13 | 21.0 [12.0, 24.0]; n=37 | 24.0 [19.5, 27.9]; n=35 | 26.5 [24.8, 31.5]; n=4 | 89 |  |
| Circulatory-arrest duration (min) — Ascending-aortic replacement only / other recorded category | 12.0 [8.0, 13.0]; n=9 | 12.0 [12.0, 13.0]; n=5 | NA; n=0 | 15.0 [11.0, 19.0]; n=2 | 11.0 [9.0, 13.0]; n=2 | 9 |  |
| Operation duration (min) — Aortic valve and/or sinus repair | 395.0 [345.0, 430.0]; n=168 | 390.0 [330.0, 425.0]; n=69 | 370.0 [335.0, 443.8]; n=12 | 372.5 [353.8, 420.0]; n=24 | 400.0 [372.5, 432.6]; n=63 | 168 |  |
| Operation duration (min) — Bentall | 452.5 [396.2, 480.0]; n=90 | 470.0 [445.0, 520.0]; n=13 | 412.5 [385.0, 480.0]; n=38 | 455.0 [422.5, 475.0]; n=35 | 467.5 [430.1, 517.5]; n=4 | 90 |  |
| Operation duration (min) — Ascending-aortic replacement only / other recorded category | 315.0 [297.5, 371.2]; n=10 | 320.0 [295.0, 385.0]; n=5 | NA; n=0 | 305.0 [292.5, 317.5]; n=2 | 310.0 [307.5, 357.5]; n=3 | 10 |  |

**Panel D. Intraoperative plasma**

| **Variable / level** | **Overall** | **G1** | **G2** | **G3** | **G4** | **Observed n** | **Global P** |
| --- | --- | --- | --- | --- | --- | --- | --- |
| Intraoperative plasma amount (mL), all patients — median [Q1, Q3] | 600 [0, 800] | 600 [0, 800] | 600 [0, 800] | 700 [600, 800] | 800 [0, 800] | 268 | 0.108 |
| Intraoperative plasma amount (mL), all patients — n observed | 268 | 87 | 50 | 61 | 70 | 268 |  |

Continuous-variable global P values use Kruskal–Wallis tests. Categorical global P values use Pearson chi-square when all expected cell counts are at least five and otherwise Fisher–Freeman–Halton Monte Carlo testing with 200,000 replicates. All percentages use variable-specific observed denominators. G1, moderate-stable; G2, low-declining; G3, preserved-rapidly-declining; G4, high-preserved. Arch procedure was classified in 267 patients (243 TAR, 23 HAR, one no arch replacement); one remained indeterminate. Circulatory-arrest duration was available for 263 patients. Arrest temperature was available for 240 patients; among the remaining 28, 25 had documented circulatory arrest without a qualifying temperature, two had unresolved arrest status, and one had documented no arrest. Structured CTA anatomy was available in 109 of 268; primary-entry-tear location in 263 of 268.

**Supplementary Table S11. Preoperative documentation of shock, tamponade, and malperfusion by PTA trajectory group.**

| **Variable / documentation category** | **Overall** | **G1** | **G2** | **G3** | **G4** | **Observed n** |
| --- | --- | --- | --- | --- | --- | --- |
| Preoperative shock — documented present | 7/268 (2.6%) | 2/87 (2.3%) | 2/50 (4.0%) | 3/61 (4.9%) | 0/70 (0.0%) | 268 |
| Preoperative shock — documented absent | 0/268 (0.0%) | 0/87 (0.0%) | 0/50 (0.0%) | 0/61 (0.0%) | 0/70 (0.0%) | 268 |
| Preoperative shock — ambiguous documentation | 9/268 (3.4%) | 7/87 (8.0%) | 1/50 (2.0%) | 1/61 (1.6%) | 0/70 (0.0%) | 268 |
| Preoperative shock — not documented | 252/268 (94.0%) | 78/87 (89.7%) | 47/50 (94.0%) | 57/61 (93.4%) | 70/70 (100.0%) | 268 |
| Preoperative shock — clinical syndrome | 7/268 (2.6%) | 2/87 (2.3%) | 2/50 (4.0%) | 3/61 (4.9%) | 0/70 (0.0%) | 268 |
| Preoperative shock — radiographic perfusion abnormality | 0/268 (0.0%) | 0/87 (0.0%) | 0/50 (0.0%) | 0/61 (0.0%) | 0/70 (0.0%) | 268 |
| Preoperative shock — anatomical involvement only | 0/268 (0.0%) | 0/87 (0.0%) | 0/50 (0.0%) | 0/61 (0.0%) | 0/70 (0.0%) | 268 |
| Preoperative shock — ambiguous | 9/268 (3.4%) | 7/87 (8.0%) | 1/50 (2.0%) | 1/61 (1.6%) | 0/70 (0.0%) | 268 |
| Preoperative shock — none documented | 252/268 (94.0%) | 78/87 (89.7%) | 47/50 (94.0%) | 57/61 (93.4%) | 70/70 (100.0%) | 268 |
| Cardiac tamponade — documented present | 7/268 (2.6%) | 2/87 (2.3%) | 2/50 (4.0%) | 0/61 (0.0%) | 3/70 (4.3%) | 268 |
| Cardiac tamponade — documented absent | 0/268 (0.0%) | 0/87 (0.0%) | 0/50 (0.0%) | 0/61 (0.0%) | 0/70 (0.0%) | 268 |
| Cardiac tamponade — ambiguous documentation | 10/268 (3.7%) | 3/87 (3.4%) | 3/50 (6.0%) | 3/61 (4.9%) | 1/70 (1.4%) | 268 |
| Cardiac tamponade — not documented | 251/268 (93.7%) | 82/87 (94.3%) | 45/50 (90.0%) | 58/61 (95.1%) | 66/70 (94.3%) | 268 |
| Cardiac tamponade — clinical syndrome | 7/268 (2.6%) | 2/87 (2.3%) | 2/50 (4.0%) | 0/61 (0.0%) | 3/70 (4.3%) | 268 |
| Cardiac tamponade — radiographic perfusion abnormality | 0/268 (0.0%) | 0/87 (0.0%) | 0/50 (0.0%) | 0/61 (0.0%) | 0/70 (0.0%) | 268 |
| Cardiac tamponade — anatomical involvement only | 0/268 (0.0%) | 0/87 (0.0%) | 0/50 (0.0%) | 0/61 (0.0%) | 0/70 (0.0%) | 268 |
| Cardiac tamponade — ambiguous | 10/268 (3.7%) | 3/87 (3.4%) | 3/50 (6.0%) | 3/61 (4.9%) | 1/70 (1.4%) | 268 |
| Cardiac tamponade — none documented | 251/268 (93.7%) | 82/87 (94.3%) | 45/50 (90.0%) | 58/61 (95.1%) | 66/70 (94.3%) | 268 |
| Coronary malperfusion — documented present | 0/268 (0.0%) | 0/87 (0.0%) | 0/50 (0.0%) | 0/61 (0.0%) | 0/70 (0.0%) | 268 |
| Coronary malperfusion — documented absent | 0/268 (0.0%) | 0/87 (0.0%) | 0/50 (0.0%) | 0/61 (0.0%) | 0/70 (0.0%) | 268 |
| Coronary malperfusion — ambiguous documentation | 0/268 (0.0%) | 0/87 (0.0%) | 0/50 (0.0%) | 0/61 (0.0%) | 0/70 (0.0%) | 268 |
| Coronary malperfusion — not documented | 268/268 (100.0%) | 87/87 (100.0%) | 50/50 (100.0%) | 61/61 (100.0%) | 70/70 (100.0%) | 268 |
| Coronary malperfusion — clinical syndrome | 0/268 (0.0%) | 0/87 (0.0%) | 0/50 (0.0%) | 0/61 (0.0%) | 0/70 (0.0%) | 268 |
| Coronary malperfusion — radiographic perfusion abnormality | 0/268 (0.0%) | 0/87 (0.0%) | 0/50 (0.0%) | 0/61 (0.0%) | 0/70 (0.0%) | 268 |
| Coronary malperfusion — anatomical involvement only | 0/268 (0.0%) | 0/87 (0.0%) | 0/50 (0.0%) | 0/61 (0.0%) | 0/70 (0.0%) | 268 |
| Coronary malperfusion — ambiguous | 0/268 (0.0%) | 0/87 (0.0%) | 0/50 (0.0%) | 0/61 (0.0%) | 0/70 (0.0%) | 268 |
| Coronary malperfusion — none documented | 268/268 (100.0%) | 87/87 (100.0%) | 50/50 (100.0%) | 61/61 (100.0%) | 70/70 (100.0%) | 268 |
| Cerebral malperfusion — documented present | 0/268 (0.0%) | 0/87 (0.0%) | 0/50 (0.0%) | 0/61 (0.0%) | 0/70 (0.0%) | 268 |
| Cerebral malperfusion — documented absent | 0/268 (0.0%) | 0/87 (0.0%) | 0/50 (0.0%) | 0/61 (0.0%) | 0/70 (0.0%) | 268 |
| Cerebral malperfusion — ambiguous documentation | 3/268 (1.1%) | 0/87 (0.0%) | 0/50 (0.0%) | 1/61 (1.6%) | 2/70 (2.9%) | 268 |
| Cerebral malperfusion — not documented | 265/268 (98.9%) | 87/87 (100.0%) | 50/50 (100.0%) | 60/61 (98.4%) | 68/70 (97.1%) | 268 |
| Cerebral malperfusion — clinical syndrome | 0/268 (0.0%) | 0/87 (0.0%) | 0/50 (0.0%) | 0/61 (0.0%) | 0/70 (0.0%) | 268 |
| Cerebral malperfusion — radiographic perfusion abnormality | 0/268 (0.0%) | 0/87 (0.0%) | 0/50 (0.0%) | 0/61 (0.0%) | 0/70 (0.0%) | 268 |
| Cerebral malperfusion — anatomical involvement only | 73/268 (27.2%) | 23/87 (26.4%) | 17/50 (34.0%) | 15/61 (24.6%) | 18/70 (25.7%) | 268 |
| Cerebral malperfusion — ambiguous | 3/268 (1.1%) | 0/87 (0.0%) | 0/50 (0.0%) | 1/61 (1.6%) | 2/70 (2.9%) | 268 |
| Cerebral malperfusion — none documented | 192/268 (71.6%) | 64/87 (73.6%) | 33/50 (66.0%) | 45/61 (73.8%) | 50/70 (71.4%) | 268 |
| Visceral/mesenteric malperfusion — documented present | 0/268 (0.0%) | 0/87 (0.0%) | 0/50 (0.0%) | 0/61 (0.0%) | 0/70 (0.0%) | 268 |
| Visceral/mesenteric malperfusion — documented absent | 0/268 (0.0%) | 0/87 (0.0%) | 0/50 (0.0%) | 0/61 (0.0%) | 0/70 (0.0%) | 268 |
| Visceral/mesenteric malperfusion — ambiguous documentation | 2/268 (0.7%) | 1/87 (1.1%) | 1/50 (2.0%) | 0/61 (0.0%) | 0/70 (0.0%) | 268 |
| Visceral/mesenteric malperfusion — not documented | 266/268 (99.3%) | 86/87 (98.9%) | 49/50 (98.0%) | 61/61 (100.0%) | 70/70 (100.0%) | 268 |
| Visceral/mesenteric malperfusion — clinical syndrome | 0/268 (0.0%) | 0/87 (0.0%) | 0/50 (0.0%) | 0/61 (0.0%) | 0/70 (0.0%) | 268 |
| Visceral/mesenteric malperfusion — radiographic perfusion abnormality | 0/268 (0.0%) | 0/87 (0.0%) | 0/50 (0.0%) | 0/61 (0.0%) | 0/70 (0.0%) | 268 |
| Visceral/mesenteric malperfusion — anatomical involvement only | 56/268 (20.9%) | 15/87 (17.2%) | 15/50 (30.0%) | 12/61 (19.7%) | 14/70 (20.0%) | 268 |
| Visceral/mesenteric malperfusion — ambiguous | 2/268 (0.7%) | 1/87 (1.1%) | 1/50 (2.0%) | 0/61 (0.0%) | 0/70 (0.0%) | 268 |
| Visceral/mesenteric malperfusion — none documented | 210/268 (78.4%) | 71/87 (81.6%) | 34/50 (68.0%) | 49/61 (80.3%) | 56/70 (80.0%) | 268 |
| Renal malperfusion — documented present | 1/268 (0.4%) | 0/87 (0.0%) | 1/50 (2.0%) | 0/61 (0.0%) | 0/70 (0.0%) | 268 |
| Renal malperfusion — documented absent | 0/268 (0.0%) | 0/87 (0.0%) | 0/50 (0.0%) | 0/61 (0.0%) | 0/70 (0.0%) | 268 |
| Renal malperfusion — ambiguous documentation | 4/268 (1.5%) | 1/87 (1.1%) | 0/50 (0.0%) | 2/61 (3.3%) | 1/70 (1.4%) | 268 |
| Renal malperfusion — not documented | 263/268 (98.1%) | 86/87 (98.9%) | 49/50 (98.0%) | 59/61 (96.7%) | 69/70 (98.6%) | 268 |
| Renal malperfusion — clinical syndrome | 0/268 (0.0%) | 0/87 (0.0%) | 0/50 (0.0%) | 0/61 (0.0%) | 0/70 (0.0%) | 268 |
| Renal malperfusion — radiographic perfusion abnormality | 1/268 (0.4%) | 0/87 (0.0%) | 1/50 (2.0%) | 0/61 (0.0%) | 0/70 (0.0%) | 268 |
| Renal malperfusion — anatomical involvement only | 68/268 (25.4%) | 20/87 (23.0%) | 16/50 (32.0%) | 14/61 (23.0%) | 18/70 (25.7%) | 268 |
| Renal malperfusion — ambiguous | 4/268 (1.5%) | 1/87 (1.1%) | 0/50 (0.0%) | 2/61 (3.3%) | 1/70 (1.4%) | 268 |
| Renal malperfusion — none documented | 195/268 (72.8%) | 66/87 (75.9%) | 33/50 (66.0%) | 45/61 (73.8%) | 51/70 (72.9%) | 268 |
| Limb malperfusion — documented present | 1/268 (0.4%) | 0/87 (0.0%) | 1/50 (2.0%) | 0/61 (0.0%) | 0/70 (0.0%) | 268 |
| Limb malperfusion — documented absent | 0/268 (0.0%) | 0/87 (0.0%) | 0/50 (0.0%) | 0/61 (0.0%) | 0/70 (0.0%) | 268 |
| Limb malperfusion — ambiguous documentation | 4/268 (1.5%) | 1/87 (1.1%) | 2/50 (4.0%) | 1/61 (1.6%) | 0/70 (0.0%) | 268 |
| Limb malperfusion — not documented | 263/268 (98.1%) | 86/87 (98.9%) | 47/50 (94.0%) | 60/61 (98.4%) | 70/70 (100.0%) | 268 |
| Limb malperfusion — clinical syndrome | 1/268 (0.4%) | 0/87 (0.0%) | 1/50 (2.0%) | 0/61 (0.0%) | 0/70 (0.0%) | 268 |
| Limb malperfusion — radiographic perfusion abnormality | 0/268 (0.0%) | 0/87 (0.0%) | 0/50 (0.0%) | 0/61 (0.0%) | 0/70 (0.0%) | 268 |
| Limb malperfusion — anatomical involvement only | 2/268 (0.7%) | 0/87 (0.0%) | 1/50 (2.0%) | 0/61 (0.0%) | 1/70 (1.4%) | 268 |
| Limb malperfusion — ambiguous | 4/268 (1.5%) | 1/87 (1.1%) | 2/50 (4.0%) | 1/61 (1.6%) | 0/70 (0.0%) | 268 |
| Limb malperfusion — none documented | 261/268 (97.4%) | 86/87 (98.9%) | 46/50 (92.0%) | 60/61 (98.4%) | 69/70 (98.6%) | 268 |

Clinical syndromes, radiographic perfusion abnormalities, and anatomical involvement were summarized separately. No inferential P values were calculated. G1, moderate-stable; G2, low-declining; G3, preserved-rapidly-declining; G4, high-preserved. Because negative documentation was not systematic in the retrospective records, these frequencies describe the available documentation only and should not be interpreted as prevalence estimates.
